# Longitudinal data analysis for rare variants detection with penalized quadratic inference function

**DOI:** 10.1038/s41598-017-00712-9

**Published:** 2017-04-05

**Authors:** Hongyan Cao, Zhi Li, Haitao Yang, Yuehua Cui, Yanbo Zhang

**Affiliations:** 1 Shanxi Medical University, Department of Health Statistics, Taiyuan, 030001 China; 2 North University of China, School of Sport and Physical Education, Taiyuan, 030051 China; 3 Hebei Medical University, Department of Epidemiology and Health Statistics, Shijiazhuang, 050017 China; 4 Michigan State University, Department of Statistics and Probability, East Lansing, MI 48824 USA

## Abstract

Longitudinal genetic data provide more information regarding genetic effects over time compared with cross-sectional data. Coupled with next-generation sequencing technologies, it becomes reality to identify important genes containing both rare and common variants in a longitudinal design. In this work, we adopted a weighted sum statistic (WSS) to collapse multiple variants in a gene region to form a gene score. When multiple genes in a pathway were considered together, a penalized longitudinal model under the quadratic inference function (QIF) framework was applied for efficient gene selection. We evaluated the estimation accuracy and model selection performance under different model settings, then applied the method to a real dataset from the Genetic Analysis Workshop 18 (GAW18). Compared with the unpenalized QIF method, the penalized QIF (pQIF) method achieved better estimation accuracy and higher selection efficiency. The pQIF remained optimal even when the working correlation structure was mis-specified. The real data analysis identified one important gene, angiotensin II receptor type 1 (AGTR1), in the Ca2+/AT-IIR/α-AR signaling pathway. The estimated effect implied that AGTR1 may have a protective effect for hypertension. Our pQIF method provides a general tool for longitudinal sequencing studies involving large numbers of genetic variants.

## Introduction

Longitudinal data are often observed in biomedical studies with repeated measures of the same subject over time. Coupled with the recent development of genome-wide SNP genotyping and next-generation sequencing technologies, longitudinal designs are becoming popular in genetic association studies because of their increased power over cross-sectional designs to detect genetic associations. Most longitudinal genetic association analyses have focused on the evaluation of associations at common variants^1–3^, which only explained a part of disease heritability^4^. Next-generation sequencing technologies provide the opportunity to study the role of rare variants in complex diseases, as evidenced by reports in the literature^5, 6^.

Because of the extremely low minor allele frequency (MAF) of rare variants (defined as variants with a MAF < 5%), the commonly-used single-variant association analysis is underpowered^7, 8^. Methods for detecting rare variants have been developed and can be broadly classified into three categories: (1) burden tests, for example, the weighted sum statistic (WSS) methods^9^; (2) variance component-based tests represented by the sequence kernel association test (SKAT)^10^; and (3) dimension-reduction based tests such as functional principal components analysis (FPCA)^11^ and the adaptive ridge regression method^12^. For a comprehensive review of the design and analysis of rare variants in association studies, please refer to Lee *et al*.^8^.

All the rare variants association tests methods described above are available for phenotypes measured at a single time point. Very few methods have been developed or extended to detect rare variants associated with longitudinal disease traits^13–16^. Yan *et al*.^15^ extended the kernel machine method to handle longitudinal continuous trait from family samples in the linear mixed model (LM) framework. Chien *et al*.^16^ extended burden test and kernel association tests to genetic longitudinal studies in the generalized estimating equations (GEE) framework. Wu *et al*.^14^ and Chiu *et al*.^13^ summarized the rare variants longitudinal studies, where most of the statistical models were based on GEE and LM models. These methods face computational challenges with limited sample size and missing data. Furthermore, large numbers of gene variables increase the complexity of modeling, especially because most genes have no association with the studied disease trait. The challenge particularly increases when the number of genes or SNP markers becomes larger than the number of samples. As such, the classical methods faces estimation instability issues when the number of variants is large. This motivates us to adopt a penalized regression method for better parameter estimation, and achieving gene selection in the mean time.

When a large number of gene variables are modelled simultaneously in a regression model, high-dimensional variable selection strategies become essential for a genetic association study. Variable selection methods with a univariate response in linear and generalized linear regression analyses have been studied extensively^17–19^. Various penalty functions have been developed for different purposes, such as the group LASSO for selecting a group of variables^20^ and the network-constrained penalty for selecting variables with a graph structure^21^. In fact, penalized regression methods have been applied to rare variants association analysis when a univariate disease trait is considered^22–24^.

For multivariate responses in a longitudinal study, variable selection methods have also been developed. Under the population-averaged (marginal) model framework, several variable selection methods have been developed for a diverging number of parameters. For example, the penalized generalized estimating equations (pGEE) method developed by Wang *et al*.^25^ can handle cases where the number of covariates have the same order as the number of individuals. Cho and Qu^26^ proposed the penalized quadratic inference function (pQIF) method for a diverging number of regression parameters, and showed that pQIF outperformed pGEE in various settings. Given the improved performance of a longitudinal design in identifying genetic variants, it is essential to develop a variable selection strategy to improve estimation accuracy and gene selection efficiency in a longitudinal study. In this work, we extended the pQIF method to a longitudinal genetic association study with rare variants and evaluated its performance with extensive simulation studies. Practical suggestions were obtained for real applications. We further applied the pQIF method to a hypertension dataset from the Genetic Analysis Workshop 18 (GAW18)^27^ and focused on the Ca2+/AT-IIR/α-AR signaling pathway to identify genes associated with the development of hypertension.

In statistical methods section, we briefly describe the quadratic inference function (QIF) method for longitudinal data, then describe the pQIF method. We then review the methods used for rare variants detection, focusing on the collapsing method that was applied in this work. The simulation studies are reported in simulation study section. In real data analysis section, we describe the application of the pQIF method to a real dataset focusing on the Ca2+/AT-IIR/α-AR signaling pathway, followed by a discussion section.

## Statistical Methods

### QIF in longitudinal data analysis

For repeated outcome or exposure measurements under a longitudinal design, the model can be expressed as:

1 $$E({Y}_{it})={\mu }_{i}\,{\rm{and}}\,g({\mu }_{i})={X}_{it}^{^{\prime} }\beta ,$$

where *Y*_*it*_ is the phenotype measured for subject *i*(*i* = 1, …, *n*) at time *t*(*t* = 1, …, *T*); *g*(·) is a known link function (i.e., identity link for continuous *Y* and logistic link for binary *Y*); *X*_*it*_ contains both time-varying covariates and time-invariant genetic variants; and *β* represents unknown coefficients. In this study, we focused our analysis on a longitudinal binary disease trait.

It is generally difficult to specify a full likelihood function for the correlated responses *Y*_*it*_. GEE is a classic population-averaged (marginal) model that requires only a working correlation for *Y*_*it*_ to be specified in order to obtain consistent estimates for the mean parameters, even when the correlation structure is mis-specified^28^. However, the estimator of the regression parameter is inefficient under mis-specification of the correlation structure^29^. The quasi-score equation in GEE is defined as:

2 $$S(\beta )=\sum {\dot{\mu }}_{i}^{T}(\beta ){V}_{i}^{-1}\{{Y}_{i}-{\mu }_{i}(\beta )\}=0,$$

where ${V}_{i}={A}_{i}^{\frac{1}{2}}{R}_{i}(\rho ){A}_{i}^{\frac{1}{2}}$ with *A*_*i*_ being a diagonal matrix of marginal variances for *Y*_*i*_, and *R*(*ρ*) being a common working correlation with nuisance parameter, the intra-class correlation coefficient *ρ*, ${\dot{\mu }}_{i}=\partial {\mu }_{i}/\partial {\beta }_{j}$. The equation can be simplified by specifying a specific correlation structure for *R*_*i*_, such as independent, exchangeable, or AR(1).

The GEE method has the advantage that the estimators of the regression coefficients are consistent even when the correlation structure is mis-specified, given that *ρ* is consistently estimated. When such a consistent estimator does not exist, Qu *et al*.^29^ suggested that the inverse of *R*(*ρ*) can be represented by a linear combination of a class of basis matrices such as *R* ^−1^(*ρ*) ≈ *a*_0_ *I* + *a*_1_ *M*_1_+, …+*a*_*S*_ *M*_*S*_, where *I* is an identify matrix and *M*_1_, …, *M*_*S*_ are known symmetric matrices. Under the QIF framework of Qu *et al*.^29^, we defined the score functions as:

3 $${\psi }_{i}(\beta )=(\begin{array}{c}{\dot{\mu }}_{i}^{T}{A}_{i}^{-1}({Y}_{i}-{\mu }_{i})\\ {\dot{\mu }}_{i}^{T}{A}_{i}^{-1/2}{M}_{1}{A}_{i}^{-1/2}({Y}_{i}-{\mu }_{i})\\ \vdots \\ {\dot{\mu }}_{i}^{T}{A}_{i}^{-1/2}{M}_{S}{A}_{i}^{-1/2}({Y}_{i}-{\mu }_{i})\end{array})$$

and the mean vector as ${\bar{\psi }}_{n}(\beta )=\frac{1}{n}{\sum }_{i=1}^{n}{\psi }_{i}(\beta )$. Then, the estimate $\hat{\beta }$ can be obtained by minimizing the QIF as

4 $${Q}_{n}(\beta )={\bar{\psi }}_{n}^{T}(\beta ){\bar{C}}_{n}^{-1}{\bar{\psi }}_{n}(\beta ),$$

where ${\bar{C}}_{n}=1/n{\sum }_{i=1}^{n}{\psi }_{i}(\beta ){\psi }_{i}^{T}(\beta )$ is a consistent estimator for Ω = var (*ψ*_*i*_), i.e.,

5 $$\hat{\beta }=\text{arg}{\min }_{\beta }{Q}_{n}(\beta ).$$

Under certain conditions, the estimation consistency of the estimator $\hat{\beta }$ as well as the asymptotic normality can be established.

### pQIF method

QIF was extended to a high-dimensional regression setup where the number of predictors can be large. The pQIF is based on the non-convex SCAD penalty given by:

6 $${p}_{{\lambda }_{n}}^{{}^{^{\prime} }}(\theta )={\lambda }_{n}\{I(\theta \le {\lambda }_{n})+\frac{{(a{\lambda }_{n}-\theta )}_{+}}{(a-1){\lambda }_{n}}I(\theta  > {\lambda }_{n})\}$$

for some *a* > 2 and *θ* > 2. The SCAD penalty function can select variables and estimate coefficients simultaneously, and possesses an “oracle property”^18^. For pQIF, the penalized score function is defined as:

7 $${U}_{n}(\beta )={Q}_{n}(\beta )+n\sum _{j=1}^{p}{p}_{{\lambda }_{n}}(|{\beta }_{j}|).$$

Because the SCAD penalty function is non-convex, the penalized score function in equation (7) can be approximated by the local quadratic approximation as follows:

8 $$\begin{array}{ll}{Q}_{n}({\beta }^{(k)})+\nabla {Q}_{n}{({\beta }^{(k)})}^{T}({\beta }_{s}-{{\beta }_{s}}^{(k)}) & +\frac{1}{2}{({\beta }_{s}-{{\beta }_{s}}^{(k)})}^{T}{\nabla }^{2}{Q}_{n}({\beta }^{(k)})({\beta }_{s}-{{\beta }_{s}}^{(k)})\\  & +\frac{1}{2}n{\beta }_{s}^{T}\prod ({\beta }^{(k)}){\beta }_{s},\end{array}$$

where ${{\beta }_{s}}^{(k)}$ is the *k*th iteration of the non-zero components. $\nabla {Q}_{n}({\beta }^{(k)})$ and ${\nabla }^{2}{Q}_{n}({\beta }^{(k)})$ are the first and second derivatives of *Q*_*n*_(*β* ^(*k*)^), and

9 $$\prod ({\beta }^{(k)})=diag\{{p}_{{\lambda }_{n}}^{^{\prime} }(|{\beta }_{1}^{(k)}|)/|{\beta }_{1}^{(k)}|,\cdots ,{p}_{{\lambda }_{n}}^{^{\prime} }(|{\beta }_{qk}^{(k)}|)/|{\beta }_{qk}^{(k)}|\}.$$

The Newton-Raphson algorithm can be applied to get ${{\beta }_{s}}^{(k+1)}$, the (*k* + 1)th iteration of the non-zero component ${{\beta }_{s}}^{(k+1)}$.

The performance of model selection in pQIF relies on the appropriate selection of the tuning parameters. The tuning parameters were chosen with the Bayesian information QIF criterion (BIQIF) which is analogous to the Bayesian information criterion and is based on QIF as the objective function. The BIQIF is defined as:

10 $$BIQI{F}_{{\lambda }_{n}}={Q}_{n}({\hat{\beta }}_{{\lambda }_{n}})+d{f}_{{\lambda }_{n}}\,\mathrm{log}(n),$$

where ${\hat{\beta }}_{{\lambda }_{n}}$ is the marginal regression parameters estimated by minimizing *U*_*n*_(*β*) in equation (7) for a given *λ*_*n*_, and *df*_*λn*_ is the number of non-zero coefficients in ${\hat{\beta }}_{{\lambda }_{n}}$. We chose the optimal tuning parameter *λ*_*n*_ which minimizes $BIQI{F}_{{\lambda }_{n}}$ in equation (10).

### Statistical methods for rare variants analysis

Rare variants association studies typically focus on multiple variants in a specific genomic region (e.g., a gene) rather than on individual variants separately. The gene- or region-based methods can be broadly categorized into three classes: (1) burden tests, (2) variance component-based tests, and (3) dimension-reduction based tests. Burden tests simply collapse multiple variants into a single genetic score^30^. For example, the cohort allelic sum test (CAST)^31^ collapses multiple rare variants into one binary variable, which indicates whether there are any rare variants. Morris and Zeggini^32^ extended CAST by counting the total number of minor alleles. The combined multivariate and collapsing method^33^ first collapses the variants into several subgroups based on some predefined criteria (e.g., allele frequencies), and then performs a multivariate test. The WSS method weights all variants differently when determining the genetic load of an individual. So, by weighting the signals from each variant, the WSS accentuates variants that are rare in an individual^9^. The variable threshold method^34^ selects the optimal rare frequency threshold on a grid of points, and estimates the p-value by a permutation procedure. All these burden tests assume all the variants share the same effect direction and magnitude (after incorporating weights). Thus, any violation of this assumption can result in a loss of power^8, 10, 35^. To overcome the limitations of the burden tests, the data-adaptive sum test (aSum) was proposed^36^. Specifically, the aSum method first estimates the direction of effect for each variant using a marginal regression model, then it changes the coding of variants accordingly, and finally uses the same method as the burden test to test for any association. However, aSum is computationally intensive because it obtains the p-value via permutations. Moreover, the estimation of regression coefficients for single rare variants is typically difficult and unstable^8^.

Variance-component based methods (e.g., SKAT^10^) assume the effect sizes of rare variants follow a normal distribution, and then test for the variance components. It has been demonstrated that burden tests were more powerful than SKAT when most of the rare variants were causal and had the same directions, whereas SKAT outperformed burden tests when the effects of rare variants were heterogeneous^37^. This motivated the development of some hybrid methods such as SKAT-O^38^ and MiST^39^, which combined the benefits of the burden tests and SKAT. These hybrid methods were more robust across a range of scenarios, but were less powerful than either one of these tests on their self-underlying assumptions^8, 30^.

Other dimension-reduction techniques are available for rare variants analysis, such as FPCA^11^ and the adaptive ridge regression method^12^. Luo *et al*.^11^ compared FPCA with seven alternative methods (including multivariate principal component analysis, WSS, and variable threshold) and concluded that, among them, FPCA was the most powerful. However, the performance of the dimension reduction techniques and variance components-based tests is not clearly known. Borrowing the idea of the WSS, we proposed to adopt the collapsing idea to collapse both rare and common variants over a gene or region into a single genetic score for further estimation and gene selection.

### WSS method with pQIF

The WSS method jointly analyses a group of SNP variants in a gene or region. Without loss of generality, here we focused on a gene to describe the method. Suppose *J* is the total number of variants in a gene. Let *G*_*ij*_ be the number of disease alleles for variant *j* in individual *i*, and *G*_*ij*_ = 0, 1, 2 under an additive genetic model. Then, each individual is scored by a single weighted average of the number of alleles in a given gene as:

11 $${C}_{i}=\sum _{j=1}^{J}{w}_{j}{G}_{ij},$$

where *w*_*j*_ is the weight given as the inverse of the standard deviation for the minor allele, i.e., ${w}_{j}=1/\sqrt{{p}_{j}(1-{p}_{j})}$ where *p*_*j*_ is the MAF of variant *j*. This weighting function assumes that rare variants have larger effect sizes than common variants^9^. A weighted gene score can be obtained for each gene. The gene-based scores are then fitted into the pQIF model to select the genes associated with a longitudinal disease trait.

After collapsing multiple (common and rare) variants in each gene with the weighted sum, the longitudinal model can be defined as:

12 $$E({Y}_{it})={\mu }_{it},\,g({\mu }_{it})={\alpha }_{0}+\sum _{k=1}^{K}{\gamma }_{k}{E}_{ikt}+\sum _{j=1}^{{p}_{n}}{\beta }_{j}{C}_{ij},$$

where *E*_*kt*_ is the *k*th time-varying or time-invariant environmental variable and *C*_*j*_(*j* = 1, …, *p*_*n*_) is the weighted sum score for the *j*th gene, which is time-invariant. This mean model is then fitted with the pQIF method for further estimation and gene selection.

### Unbalanced data implementation for pQIF

In a real longitudinal study, unbalanced data, which are featured as cluster sizes that vary for different individuals, are commonplace because of missing data. In such cases, a transformation matrix *H*_*i*_ can be applied for each subject to fit the pQIF model^26^. For each fully observed individual without missing data, *H*_*i*_ is expressed as an *m* × *m* identity matrix for the *i*th subject, where *m* is the total repeated time point. For the *i*th subject with missing measurements, *H*_*i*_ is generated by deleting the columns that correspond to the missing measurements. Consider a study with a total of three time points. For an individual *i* with the 3^rd^ time point missing, the transformation matrix *H*_*i*_ can be expressed as $${H}_{i}=[\begin{array}{cc}1 & 0\\ 0 & 1\\ 0 & 0\end{array}]$$. Otherwise *H*_*i*_ = *I*_3×3_ if no measurements are missing. Then *ψ*_*i*_(*β*) in equation (3) can be replaced by:

13 $${\psi }_{i}^{\ast }(\beta )=(\begin{array}{c}{({\dot{\mu }}_{i}^{\ast })}^{T}{({A}_{i}^{\ast })}^{-1}({Y}_{i}^{\ast }-{\mu }_{i}^{\ast })\\ {({\dot{\mu }}_{i}^{\ast })}^{T}{({A}_{i}^{\ast })}^{-1/2}{M}_{1}{({A}_{i}^{\ast })}^{-1/2}({Y}_{i}^{\ast }-{\mu }_{i}^{\ast })\\ \vdots \\ {({\dot{\mu }}_{i}^{\ast })}^{T}{({A}_{i}^{\ast })}^{-1/2}{M}_{S}{({A}_{i}^{\ast })}^{-1/2}({Y}_{i}^{\ast }-{\mu }_{i}^{\ast })\end{array}),$$

where ${\dot{\mu }}_{i}^{\ast }={H}_{i}{\dot{\mu }}_{i}$, ${\mu }_{i}^{\ast }={H}_{i}{\mu }_{i}$, ${Y}_{i}^{\ast }={H}_{i}{Y}_{i}$, ${A}_{i}^{\ast }={H}_{i}{A}_{i}{H}_{i}^{T}$. This leads to a transformed mean vector ${\bar{\psi }}_{n}^{\ast }(\beta )=1/n{\sum }_{i=1}^{n}{\psi }_{i}^{\ast }(\beta )$ for further pQIF estimation with unbalanced data.

### Simulation Study

We performed extensive simulations to examine the performance of the pQIF for longitudinal sequencing association studies. We examined the pQIF under different sample sizes and different variable dimensions. The performance of the pQIF under mis-specified correlation structures was also evaluated, based on three different working correlations (independent, AR(1), and exchangeable).

The simulation was based on the GAW18 real sequencing data. The GAW18 dataset was based on a longitudinal study design consisting of whole-genome sequencing of 1043 individuals in the San Antonio Family studies with pedigrees. Among the 1043 individuals, 142 are unrelated and had both real phenotype data and imputed sequence data. The sequencing data for GAW18 were provided only for markers on odd-numbered autosomes. Two phenotype datasets were provided: one was the real phenotype data including systolic blood pressure and diastolic blood pressure along with other covariates such as current use of antihypertensive medications, sex, age, and smoking status up to four time points; the other was the simulated longitudinal phenotype data that were based on the real genotype data. Along with both datasets, a list of “functional loci” associated with the simulated phenotypes were also provided, thus the true functional mechanism is known for the simulated data.

Here we focused on the 142 unrelated individuals in both the simulation and real data analyses. In the simulations, we chose the top five influential genes provided in the GAW18 dataset, MAP4, TNN, NRF1, LEPR, and FLT3, as the true effect genes in our simulation studies. Because the sample size (142) was not large enough to demonstrate the performance of the pQIF, we bootstrapped additional samples assuming that the 142 individuals represented the population. For each bootstrapped sample, we fixed the original sequencing data, but simulated new binary longitudinal responses *Y*_*it*_ based on the following model:

14 $$\mathrm{log}\,it({\mu }_{it})={\beta }_{0}+{\beta }_{age}ag{e}_{it}+{\beta }_{smoke}smok{e}_{it}+\sum _{j=1}^{{p}_{n}}{\beta }_{j}{C}_{ij},\,t=1,\ldots ,3,$$

where *C*_*ij*_ is the weighted score for gene *j*, chosen from the above five genes. We also simulated noisy gene variants with no genetic effect. Each noisy gene consists of 10 SNP variants with the proportion of rare and common variants set as 6:4. An additive coding {0, 1, 2} for each SNP variant was used. Both the rare and common variants were collapsed over genes as a weighted score using the WSS method. Ages were taking from the original dataset, and missing age values at exams were filled in by adding or subtracting 3.9 years between exam 1 and exam 2 and 6.9 years between exam 1 and exam 3. Tobacco smoking was generated as follows: 22.9% of individuals were selected randomly to be smokers at exam 1, and 1.45% were deemed to have quit smoking during each exam. This follows the same quitting rate as in the real dataset. All the variables were standardized to have mean zero and standard deviation one before further analysis. The R package mvtBinaryEP was used to generate the longitudinal binary responses. Under each scenario, 200 simulation runs were conducted.

To evaluate the estimation accuracy, we calculated the total mean squared error (TMSE) as

15 $$TMSE=\frac{1}{200}\sum _{j=1}^{200}\parallel {\hat{\beta }}^{(j)}-\beta {\parallel }^{2}/p,$$

where *p* is the dimension of *β* and ${\hat{\beta }}^{(j)}$ is the estimated value for *β* in the *j*th simulation run. We also calculated the mean squared error (MSE) for noisy gene variants (NMSE) in the same way as we calculated TMSE. True positive (TP) and false positive (FP) rates were calculated to evaluate the model selection performance.

### Selection and estimation performance under the true correlation structure

We compared the model performance under three different sample sizes: *n* = 142 unrelated samples from the GAW18 dataset, and *n* = 250 and *n* = 500 based on the bootstrapped samples. The total number of covariates (*p*) including environmental variables and genes (both effective and noisy ones) were assumed to be 20 and 40, and the number of effective variables was assumed to be *q* = 4 and 6. Data were simulated assuming an AR(1) correlation structure and were subsequently analyzed by applying an AR(1) correlation structure (assuming the true correlation structure was known). In the first simulation scenario, the true coefficients were assumed to be *β* = (0.9, −0.7, −0.6, 0.5, 0, …, 0)^*T*^, where the nonzero coefficients corresponded to covariates age and three genes (MAP4, TNN, and NRF1). In the second scenario, *β* = (0.9, −0.7, −0.7, −0.6, −0.6, 0.5, 0, …, 0)^*T*^, where nonzero coefficients corresponded to covariate age and five genes (MAP4, TNN, LEPR, FLT3, and NRF1). Two intra-class correlation coefficients were considered with *ρ* = 0.4 in scenario 1 and *ρ* = 0.7 in scenario 2. The optimal tuning parameter *λ* was chosen by a grid search based on a sequence of 100 values of equal interval in [0.01, *λ*_max_], where *λ*_max_ is the value for which all coefficients were shrunk to zero. *λ*_max_ was set differently under different sample sizes. Here we set *λ*_max_ = 0.35, 0.25, 0.2 for sample sizes *n* = 142, 250, and 500, respectively. We set the tolerance level tol = 10^−12^ in the QIF method, and tol = 10^−10^ in the pQIF method (the tol of pQIF has to be larger than QIF) to control the FP rates in the simulation studies.

**Figure 1:**
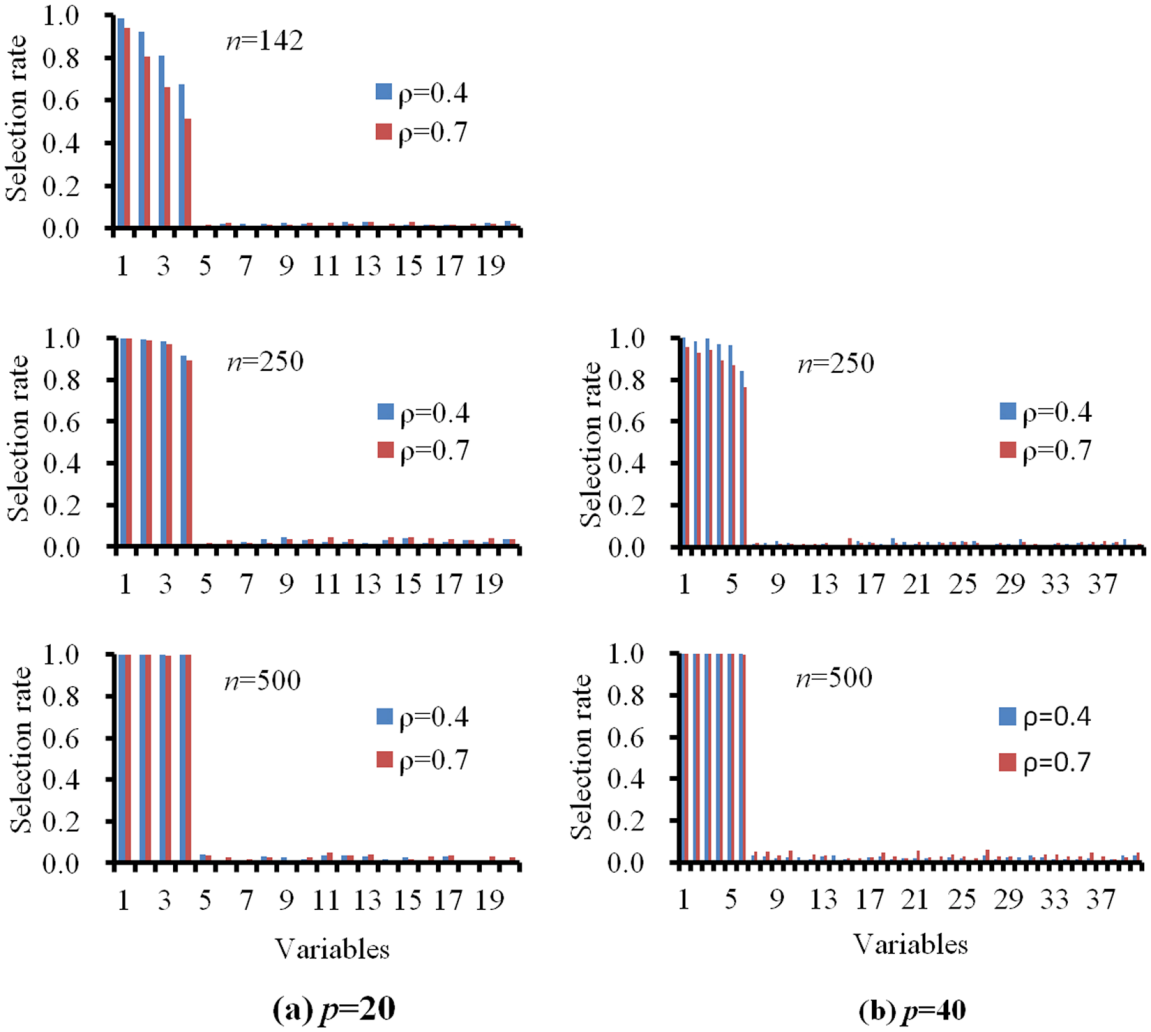
Performance of the pQIF for different sample sizes and different dimensions. (**a**) *p* = 20, (**b**) *p* = 40. The horizontal axis represents the variables, where 1 represents covariate age, 2–4 represent the three gene variables (MAP4, TNN, and NRF1) when *p* = 20, and 2–6 represent the five gene variables (MAP4, TNN, LEPR, FLT3, and NRF1) when *p* = 40, others represent the noise variables. The true and working correlation structures were set as AR(1). The title of each subfigure (e.g., “*n* = 142” in the top left panel) refers to the sample size. Since the pQIF did not converge well for *n* = 142, *p* = 40 in some simulations runs, the estimation results were not listed in the figure.

Figure 1 shows the performance of the pQIF for different sample sizes and different dimensions. When *p* = 20, the pQIF chose most of the TP variables, even when *n* = 142, and the FP selection rate was very low under different model conditions. For the increased sample sizes, the TP selection rate also increased. The TP selection rate for *ρ* = 0.4 was higher than for *ρ* = 0.7 for *n* = 142 but the difference in the TP selection rate between the two *ρ* values diminished as the sample size increased. The detailed estimation accuracy of the pQIF under different model setups is listed in Table 1. We did not list the results for *n* = 142 when *p* = 40 because the pQIF did not converge well for the larger *p* value in many simulations runs. Thus, in real applications, when the gene dimension is large, the pQIF may not be useful because of computational limitations, especially when the sample size is small.

**Table 1:** Estimation accuracy of parameters for the QIF and pQIF under different model conditions.

Sample	Method	*p* = 20				*p* = 40			
		*ρ* = 0.4		*ρ* = 0.7		*ρ* = 0.4		*ρ* = 0.7	
		TMSE^a^	NMSE^b^	TMSE	NMSE	TMSE	NMSE	TMSE	NMSE
*n* = 142	oQIF^c^	0.0068	—	0.0093	—	—	—	—	—
	QIF	0.0711	0.0523	0.2458	0.1837	—	—	—	—
	pQIF	0.0191	0.0033	0.0329	0.0056	—	—	—	—
*n* = 250	oQIF	0.0029	—	0.0043	—	0.0029	—	0.0035	—
	QIF	0.0211	0.0191	0.0305	0.0277	0.0659	0.0462	0.301	0.2033
	pQIF	0.0062	0.0022	0.0098	0.0041	0.0067	0.0020	0.0123	0.0028
*n* = 500	oQIF	0.0015	—	0.0022	—	0.0012	—	0.0017	—
	QIF	0.0076	0.0069	0.0110	0.0099	0.0123	0.0102	0.018	0.0149
	pQIF	0.0023	0.0009	0.0035	0.0015	0.0021	0.0010	0.0036	0.0020

Since the pQIF did not converge well for *n* = 142, *p* = 40 in some simulations runs, the estimation results were not listed in the table. The notation “—” indicates that the results were not available for *n* = 142, *p* = 40, and so does for the NMSE of oQIF.

^a^TMSE = total mean squared error for all the variables in the model.

^b^NMSE = mean squared error for all the noisy gene variants in the model.

^c^oQIF = Oracle QIF.

Compared to the unpenalized method, the TMSE of the penalized approach was much smaller (Table 1). As the sample size increases, the TMSE of the penalized results gets closer to the oracle one which assumes the true regression model is known. Although the correlation had little effect on selection, we found that larger error correlations led to larger TMSE and NMSE. The difference became smaller when the sample size was larger. In addition, we did not see a clear impact of variable dimension on MSE; the TMSE and NMSE were quite similar in both cases (*p* = 20 and *p* = 40).

In a short summary, when data were analyzed assuming the true covariance structure was known, the pQIF performed well with a low FP selection rate. However, if the sample size is small relative to the variable dimension, the pQIF may not converge well due to computational issues.

### Selection and estimation performance when the covariance is mis-specified

We next examined the performance of the pQIF when the covariance structure was mis-specified, under three different correlation structures: independent, AR(1), and exchangeable. Because we had already evaluated its performance with different sample sizes and different data dimensions, here we evaluated it only with *n* = 300, *p* = 20, and *ρ* = 0.5. We considered one covariate age and three gene variables, MAP4, TNN, and NRF1. The coefficients for the four variables were set as, *β* = (0.9, −0.7, −0.6, 0.5)^*T*^ and the rest were set as zero. To choose the best tuning parameter *λ*_*n*_, we set the sequence as 100 values of equal interval in [0.01, 0.25]. We simulated data under each correlation structure and analyzed data separately assuming independent, AR(1), and exchangeable correlations. Our aim was to assess the selection performance under a mis-specified working correlation.

**Figure 2:**
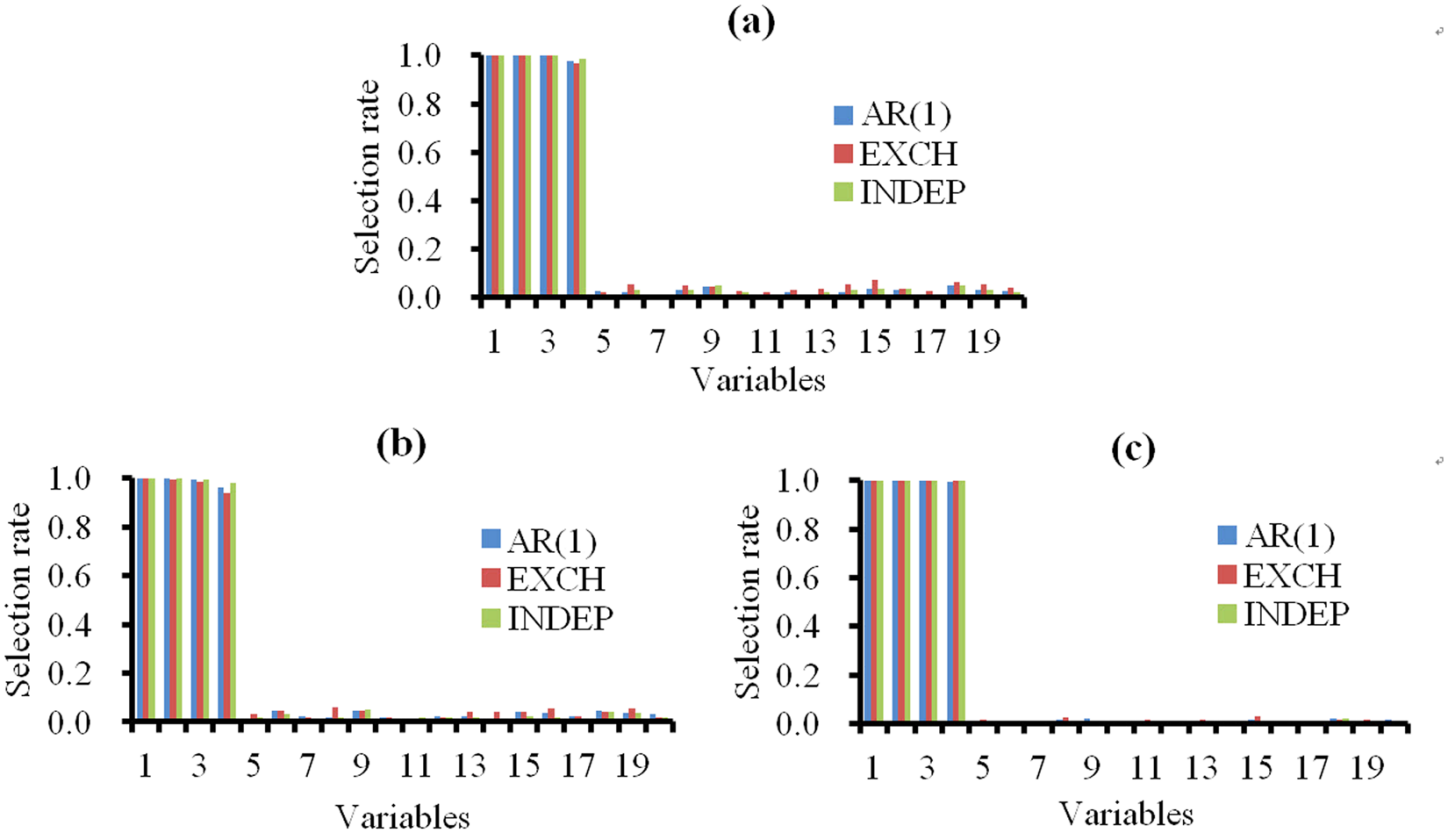
Model selection performance of the pQIF under three different working correlation structures. The true correlation structures are assumed as (**a**) AR(1) structure, (**b**) EXCH (exchangeable) structure, (**c**) INDEP (independent) structure. AR(1), EXCH (exchangeable), and INDEP (independent) in each sub-part are the working correlation structures. The bootstrapped sample size is 300, *p*=20, and *ρ*=0.5. The horizontal axis represents the variables, where 1 represents covariate age and 2–4 represent the three gene variables (MAP4, TNN, and NRF1).

Figure 2 displays the model selection performance of the pQIF under different working correlation structures. It shows that the pQIF was robust to the model mis-specification, in the sense that the selection rates for different coefficients were similar when the data were fitted assuming different covariance structures. The detailed MSEs of the pQIF under the three correlation structures are listed in Table 2. No significant differences were observed among the MSEs for different correlation structures.

**Table 2:** Estimation accuracy of the pQIF method under three types of working correlations, AR(1), EXCH (exchangeable), INDEP (independent).

True correlation	Working correlation	MSE1^a^	NMSE^b^	TMSE^c^
AR(1)	AR(1)	0.0182	0.002	0.0052
	EXCH	0.0225	0.0027	0.0067
	INDEP	0.0164	0.0018	0.0047
EXCH	AR(1)	0.0215	0.0025	0.0063
	EXCH	0.0260	0.0029	0.0075
	INDEP	0.0183	0.0019	0.0052
INDEP	AR(1)	0.0090	0.0002	0.0020
	EXCH	0.0097	0.0003	0.0022
	INDEP	0.0078	0.0001	0.0017

^a^MSE1 = mean squared error of the four nonzero coefficients for age, *MAP4*, *TNN*, and *NRF1*.

^b^NMSE = mean squared error for all the noisy gene variants in the model.

^c^TMSE = total mean squared error for all the variables in the model.

In summary, the pQIF remained optimal even when the working correlation structure was mis-specified. No significant differences in the MSEs were found when the data were analyzed assuming different correlation structures, which implies the pQIF method was robust even when the correlation structure was mis-specified.

### Real data analysis

We applied the pQIF method to the GAW18 real dataset to identify important genes associated with hypertension. We focused on the binary hypertension trait (yes = 1/no = 0) of 142 unrelated individuals, measured over four time point (133 individuals attended the 1^st^ exam, 89 attended the 2^nd^ exam, 92 attended the 3^rd^ exam, and 37 attended the 4^th^ exam). Because the 4th time point had a large number of missing values, imputation would be unreliable. Thus, we focused our analysis on the first three time points. The hypertension diagnosis was based on the criteria of systolic blood pressure >140 and diastolic blood pressure >90, or on antihypertensive medications being taken at the time of diagnosis. A description of the related phenotype data is summarized in Table 3.

**Table 3:** Distribution of age, sex, smoking, and hypertension in the GAW18 real dataset at different exam stages.

Variables	values	Exam 1	Exam 2	Exam 3
Age (years)	(20.3~96.72)	53.30 ± 15.91^a^	56.38 ± 12.96	58.24 ± 11.89
Sex	1 = Male, 2 = Female	61:81^b^	61:81	61:81
Smoking	1 = Smoking, 0 = Non-smoking	33:98	14:67	14:78
Hypertension	1 = Hypertension, 0 = Non-Hypertension	41:92	49:40	50:42

^a^ $(\bar{X}\pm S)$ for age in each exam, where $\bar{X}$ is the average and *S* is the standard deviation.

^b^Ratios for male: female, smoking: non-smoking, and hypertension: non-hypotension.

The adrenergic signaling pathway has been related to hypertension and studies have shown that large genetic variations exist in the genes involved in this pathway^40–42^. Thus, we focused our analysis on this pathway and evaluated the relationship between the involved genes and hypertension. Adrenergic signaling in cardiomyocytes was chosen from the KEGG pathway database^43^ (http://www.kegg.jp/kegg/kegg1.html). Part of the adrenergic signaling in cardiomyocytes pathway, which we defined as the Ca2+/AT-IIR/α-AR signaling pathway (see Supplementary Fig. S1). This pathway contains the three initial signals, Ca2+, AT-IIR, and α-AR, that are present on the cell membrane.

There are 16 proteins in the Ca2+/AT-IIR/α-AR signaling pathway. Different genes can encode the same proteins, for example, phospholipase C is coded by PLCB1, PLCB2, PLCB3, and PLCB4, which are located on chromosomes 20, 15, 11, and 20 respectively. Because the genetic information for even numbered chromosomes is not available in the GAW18 dataset, we excluded the genes in even numbered chromosomes in our study. We then chose one gene randomly from the remaining coding genes for each protein. Finally, we selected 11 genes in the Ca2+/AT-IIR/α-AR signaling pathway: AGTR1, ADRA1B, GNAQ, PLCB3, PRKCA, PPP1CA, CAMK2A, CALM3, RYR2, PPP2CA, and CREB3L2. Other covariates in the analysis were age, gender, and tobacco smoking. Imputation for missing ages was performed as described for the simulation study. We filled the missing smoking values with the adjacent values, or with the sample smoking probability at the corresponding time point if no smoking was recorded for that subject.

The unbalanced longitudinal binary hypertension responses were analyzed with the “transformation matrix” method for the pQIF described in unbalanced data implementation for pQIF section. The intercept was not penalized in this analysis. All the predictors were standardized to have a mean of zero and standard deviation of one.

We applied an AR(1) working correlation structure in the analysis. The gene score for each gene was collapsed with multiple rare and common variants using the WSS method. Table 4 shows the estimation of the QIF and pQIF. The pQIF selected age and AGTR1, but not tobacco smoking and gender; the other 10 genes with coefficients shrunk to zero are indicated by “—”. The QIF without penalty did not achieve sparsity, and hence did not serve the purpose of variable selection. Although one can test individual effect based on the estimated standard errors, such a test only assesses the partial effect of an individual variable while held the others constant in the model. When there exists correlation among the variables, such a test for partial effect cannot reveal the important role of a variable. However, the penalized method can fit and estimate multiple variables simultaneously in a regression model. The selection consistency and oracle property of the penalized method guarantee the importance of the selected variables with non-zero coefficients.

**Table 4:** The coefficients estimated by the QIF and pQIF methods.

Variables	QIF (S.E)^a^	pQIF
Intercept	3.438 (1.458)	0.054
Age	6.903 (2.391)	2.199
Gender	2.829 (1.082)	—
Smoking	1.170 (0.861)	—
AGTR1	−4.546 (1.697)	−0.889
ADRA1B	−0.916 (0.627)	—
GNAQ	−3.311 (1.335)	—
PLCB3	−0.008 (0.611)	—
PRKCA	−1.598 (0.953)	—
PPP1CA	1.906 (0.857)	—
CAMK2A	−0.535 (0.532)	—
CALM3	−1.593 (0.690)	—
RYR2	−0.883 (0.645)	—
PPP2CA	4.688 (1.826)	—
CREB3L2	−0.240 (0.367)	—

AGTR1, ADRA1B, GNAQ, PLCB3, PRKCA, PPP1CA, CAMK2A, CALM3, RYR2, PPP2CA, and CREB3L2 were genes in the Ca2+/AT-IIR/α-AR signaling pathway. Other covariates in the analysis were age, gender, and tobacco smoking. The notation “—” indicates that the coefficient of the related variable was penalized to 0.

^a^S.E = standard error of the coefficient estimate.

AGTR1 has 80 common variants and 215 rare variants. Because the WSS approach emphasized rare variants, the pQIF result implies that rare variants in AGTR1 may play important roles for hypertension. The negative coefficient obtained for AGTR1 indicates that the synergistic effects of multiple variants on AGTR1 are protective for hypertension by preventing elevated blood pressure due to angiotensin II. Adjusting for age, gender, and smoking effect in the pQIF model, every one unit increase in gene score of the risk variants in this gene will result in 41% decrease in the risk of hypertension. Because we analyzed only genes in odd numbered autosomes in the Ca2+/AT-IIR/α-AR signaling pathway and because we chose only one gene encoding each protein, we could have missed other important genes in this pathway. Nevertheless, our gene-based longitudinal association analysis indicates the important protective role of AGTR1 on hypertension.

## Discussion

Next-generation sequencing data are generated routinely in many laboratories in order to identify common and rare variants associated with complex diseases. With longitudinally collected disease traits, it is possible to understand disease progression as well as the underlying dynamic genetic mechanisms. However, very few studies have reported the association of rare variants with longitudinal traits, especially in a high-dimensional regression setup. In this work, we explored gene-based association studies for next-generation sequencing data with longitudinal measures of binary phenotypic traits using the pQIF method. We evaluated the performance of the pQIF method based on extensive simulation studies. The results indicated that the pQIF worked well when the sample size was relatively large but the method had convergence issues with a small sample size. The pQIF model is proposed for diverging numbers of covariates and holds for *p* = *o*(*n* ^1/4^). This might explain the poor convergence rates of the pQIF in the simulation studies when *p* = 40 and *n* = 142. Compared to the poor convergence when *p* = 40, *n* = 142 and *ρ* = 0.4 in the simulation studies, when the sample size *n* increases from 142 to 200, nearly all the 200 runs can converge. The convergence of *ρ* = 0.4 performs better than *ρ* = 0.7, indicating that the convergence is better when the intracluster correlation is low. Computation using the pQIF method is fast. For example, for *p* = 20, *n* = 142, and *ρ* = 0.4, the average running time for the pQIF in each simulation run was about 3 mins. In addition, the total MSE of the pQIF method was much smaller compared with the unpenalized QIF methods, indicating the relative gain of fitting a penalized model.

In this paper, we focused on next-generation sequencing longitudinal binary data analysis, with the special feature of dealing with rare variants, intracluster correlations, and high dimensions. Although longitudinal genetic data analyses have been reported previously, only a few of these reports focused on rare variants^13–16^. The kernel machine method based on the LM framework^15^ was extended to rare variants in longitudinal data for a family-based study, but only applicable to continuous traits. The SKAT method proposed by Chien *et al*.^16^ was built under the GEE framework and was applicable to longitudinal binary data. However, the SKAT approach fits genes one at a time, and can be computationally expensive^15^. Analyzing multiple genes simultaneously in a regression framework can greatly enhance the association study performance involving rare variants^7, 44^. With a limited sample size and a large number of genes in sequencing data, we approached the problem based on the pQIF method to conduct gene selection and estimation simultaneously for a diverging number of regression parameters. Our simulation studies provide a practical guidance to implement the method for longitudinal sequencing association studies with sequencing data.

In the real data analysis, the pQIF identified one important gene in the Ca2+/AT-IIR/α-AR signaling pathway, which further confirmed that the angiotensin II receptor protein AGTR1 had important physiological functions such as vasoconstriction, cellular proliferation, and growth^45, 46^. Hypertension is a complex and multifactorial polygenic disease. The risk loci that have been discovered so far are very limited and explain only a small part of hypertension heritability^47, 48^. Polymorphisms in AGTR1 associated with hypertension have been studied, but the results were inconsistent and conflicting^49–51^. Mottl *et al*.^47^ concluded that analyses that focused on single variants were fruitless and multiple variants analysis was needed. Our analysis improves the current approaches by integrating multiple common and rare variants in a gene (or region). However, further biological experiments are needed to verify the real biological function of the identified gene.

Although the pQIF method provides a powerful tool for analyzing longitudinal sequencing data, there are some limitations in this work. First, we describe our strategies using the WSS to collapse multiple variants in a gene region for simplicity. The WSS collapsing method, which gives a single weighted score incorporated by collapsing rare and common variants in a gene, could suffer from power loss if the assumption of same effect direction and magnitude for all variants is violated^8^. This collapsing method can be improved further by adopting more powerful strategies to detect heterogeneous effects such as the aSum statistic method. Second, given the large numbers of genetic variants in a pathway or in genome-wide data, the pQIF may be limited by the amount of data it can handle. Other methods such as the penalized participant-specific (conditional) model could be alternatives to the pQIF for longitudinal binary data. For example, Groll and Tutz^52^ proposed generalized linear mixed models by L_1_-penalized using a gradient ascent algorithm to maximize the penalized log-likelihood. Schelldorfer *et al*.^53^ proposed GLMMLasso, which can handle problems where the number of variables is in the thousand using an efficient coordinate gradient descent algorithm. Third, to further evaluate the robustness of the pQIF approach, independent longitudinal sequencing data sets should be included and tested. In addition, we did not allow the coefficients of time-invariant covariates in the pQIF to vary over time, thus changing patterns of genetic effects over time could not be captured. This can be improved by adopting a penalized varying-coefficient model under the QIF framework^54^, and will be investigated in our future work.

In conclusion, our research sheds light on the analysis of next-generation sequencing longitudinal binary data. We found that the penalized models were more efficient than the unpenalized models with interpretable regression coefficients by achieving variable selection and estimation simultaneously. The pQIF together with the collapsing methods provides a powerful tool to evaluate the synergistic effects of both rare and common variants in a gene or a genetic region with next-generation sequencing data in a longitudinal design.

## Electronic supplementary material

Supplementary Figure S1. Ca2+/AT-IIR/α-AR signaling pathway
