# Supplementary material for: Longitudinal data analysis for rare variants detection with penalized quadratic inference function
Source: Sci Rep. 2017 Apr 5;7:650. doi: 10.1038/s41598-017-00712-9 (PMC5429681; doi:10.1038/s41598-017-00712-9)
Supplement: Supplementary file 1 — Supplementary Figure S1. Ca2+/AT-IIR/α-AR signaling pathway [file 41598_2017_712_MOESM1_ESM.pdf]

Longitudinal data analysis for rare variants detection with penalized quadratic inference function

Hongyan Cao[1], Zhi Li[2], Haitao Yang[3], Yuehua Cui[1,4*], and Yanbo Zhang[1*]

[1]Shanxi Medical University, Department of Health Statistics, Taiyuan, 030001, China
[2]North University of China, School of Sport and Physical Education, Taiyuan, 030051, China
[3]Hebei Medical University, Department of Epidemiology and Health Statistics, Shijiazhuang, 050017, China
[4]Michigan State University, Department of Statistics and Probability, East Lansing, MI, 48824, USA.
* Corresponding authors: cui@stt.msu.edu, sxmuzyb@126.com

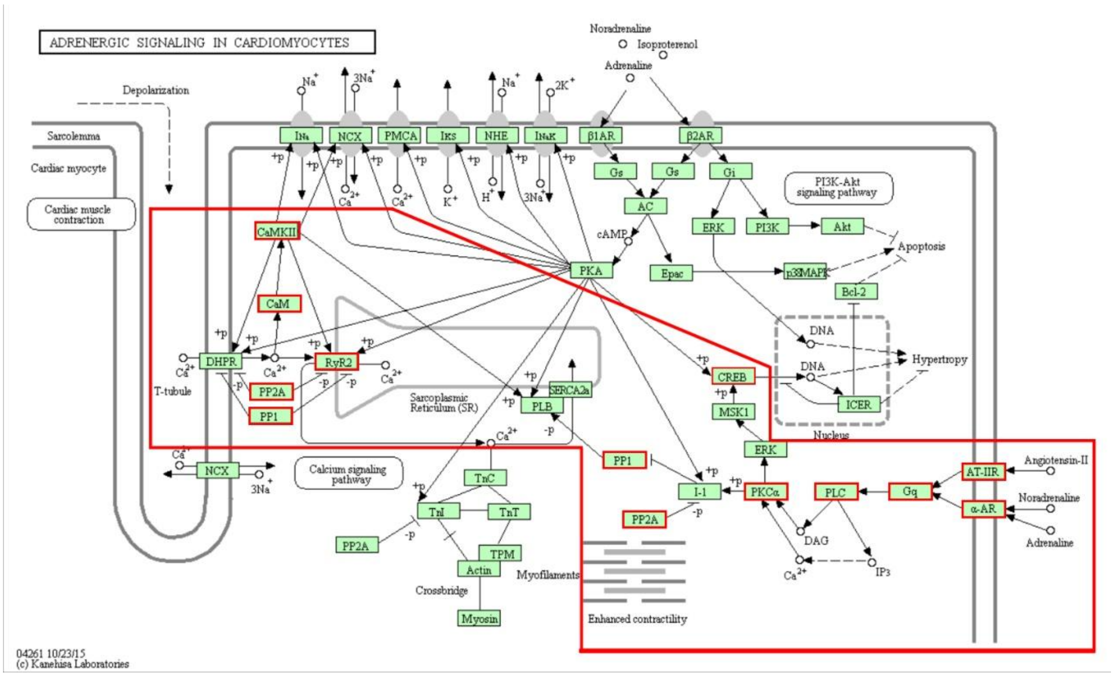

Supplementary Figure S1. Ca2+/AT-IIR/α-AR signaling pathway. The Ca2+/AT-IIR/α-AR signaling pathway marked by red frame is part of the "Adrenergic signaling in cardiomyocytes" pathway (map04261), chosen from the KEGG (Kanehisa et al., 2017) pathway database (http://www.genome.jp/dbget-bin/www_bget?map04261). The Ca2+/AT-IIR/α-AR signaling pathway was defined by three initial signals, Ca2+, AT-IIR, and α-AR, that are present on the cell membrane. The proteins in small red rectangles were included in the GAW18 real data analysis (excluding proteins coded by genes in the even numbered autosomes). Two PP1 and two PP2A proteins are involved in the Ca2+/AT-IIR/α-AR signaling pathway.

References

1. Kanehisa, M., Furumichi, M., Tanabe, M., Sato, Y. & Morishima, K. KEGG: new perspectives on genomes, pathways, diseases and drugs. *Nucleic acids research* **45,** D353-d361, doi:10.1093/nar/gkw1092 (2017).
